# Coexistent VO_2_ (M) and VO_2_ (B) Polymorphous Thin Films with Multiphase-Driven Insulator–Metal Transition

**DOI:** 10.3390/nano13091514

**Published:** 2023-04-28

**Authors:** Mengxia Qiu, Wanli Yang, Peiran Xu, Tiantian Huang, Xin Chen, Ning Dai

**Affiliations:** 1 State Key Laboratory of Infrared Physics, Shanghai Institute of Technical Physics, Chinese Academy of Sciences, Shanghai 200083, China; qiumx@shanghaitech.edu.cn (M.Q.); prxu@mail.ustc.edu.cn (P.X.); huangtiantian@mail.sitp.ac.cn (T.H.); 2 School of Physical Science and Technology, ShanghaiTech University, Shanghai 201210, China; 3 Hangzhou Institute for Advanced Study, University of Chinese Academy of Sciences, Hangzhou 310024, China; yangwanli@sitp.mail.ac.cn

**Keywords:** vanadium dioxide, polymorphs, monoclinic phase, insulator–metal transition

## Abstract

Reversible insulator–metal transition (IMT) and structure phase change in vanadium dioxide (VO_2_) remain vital and challenging with complex polymorphs. It is always essential to understand the polymorphs that coexist in desired VO_2_ materials and their IMT behaviors. Different electrical properties and lattice alignments in VO_2_ (M) and VO_2_ (B) phases have enabled the creation of versatile functional devices. Here, we present polymorphous VO_2_ thin films with coexistent VO_2_ (M) and VO_2_ (B) phases and phase-dependent IMT behaviors. The presence of VO_2_ (B) phases may induce lattice distortions in VO_2_ (M). The plane spacing of (011)_M_ in the VO_2_ (M) phase becomes widened, and the V-V and V-O vibrations shift when more VO_2_ (B) phase exists in the VO_2_ (M) matrix. Significantly, the coexisting VO_2_ (B) phases promote the IMT temperature of the polymorphous VO_2_ thin films. We expect that such coexistent polymorphs and IMT variations would help us to understand the microstructures and IMT in the desired VO_2_ materials and contribute to advanced electronic transistors and optoelectronic devices.

## 1. Introduction

Polarizable metal–oxygen bonds [1] and strong correlations among localized valance electrons in transition metal oxides [2,3] have sparked intensive interest, and they have shown promise in optoelectronics [4,5,6,7], sensors [8,9,10,11] and catalysis [12,13,14,15]. As one of the correlated electronic oxides, VO_2_ has several polymorphs, including VO_2_ (R) [16], VO_2_ (B) [17] and VO_2_ (M) [18]. Various crystalline symmetries and electronic structures in these polymorphs have shown versatile potential applications [19,20,21]. Wherein, the stable monoclinic VO_2_ (M) (*P2_1_/c*) phase has attracted widespread attention due to a reversible IMT near 340 K. Lattice variations are often connected with the transformation between delocalized and localized states during IMT in VO_2_. Meanwhile, pure electronic behaviors have been observed due to strain-engineering and lattice-freezing during IMT processes [22,23]. Companied with IMT, structural distortion generally occurs within a first-order phase change from a low-temperature monoclinic VO_2_ (M) phase to a high-temperature rutile VO_2_ (R) phase [24]. In the insulating VO_2_ (M) phase, adjacent vanadium atoms form zigzag chains with two types of vanadium–vanadium bond lengths along the *c_R_* axis, and the shorter one may form a vanadium–vanadium dimer, which makes the electrons localized [25].

The polymorphous lattices and crystalline phases often affect electrical and optical behaviors in VO_2_, which are closely related to the vanadium–vanadium bonds. Different from the VO_2_ (M) phase, the VO_2_ (B) (*C2/m*) phase is a Wadsley phase with a quasi-layered monoclinic structure [26] and undergoes IMT over a wide temperature range from 180 K to 300 K [27], while the vanadium–vanadium pairs induce layered alignments. It is noted that the edge-sharing VO_6_ octahedra in the VO_2_(B) phase aid ion diffusion and gas reduction in ion batteries and energy storage devices [28]. When VO_2_ (B)-like vanadium–vanadium bonds exist in VO_2_ (M) lattices, the microstructures, the electronic states and IMT behaviors in VO_2_ (M) may be modified due to the variations in crystal field energy and orbital occupancies near the Fermi level [29]. Actually, it is still of significance and challenging to understand microstructural changes and IMT in the coexistent polymorphs in VO_2_ towards versatile applications [30].

Herein, we described the lattice variations and IMT behaviors in VO_2_ thin films with coexistent VO_2_ (M) and VO_2_ (B) phases. The widened plane spacing of (011)_M_ and varied V-V and V-O phonon vibrations were induced by the coexisting VO_2_ (B) phase in the VO_2_ (M) thin films, as confirmed via Raman spectra and X-ray diffraction (XRD). The IMT temperature for the VO_2_ (M) thin films increased when more VO_2_ (B) phases were presented in the polymorphous thin films. We expected such phase-dependent IMT behaviors to aid our understanding of polymorphous lattice variations and electronic phase changes in correlated vanadium oxides and enable the fabrication of advanced optical, electronic and even energy devices.

## 2. Results and Discussion

**Figure 1:**
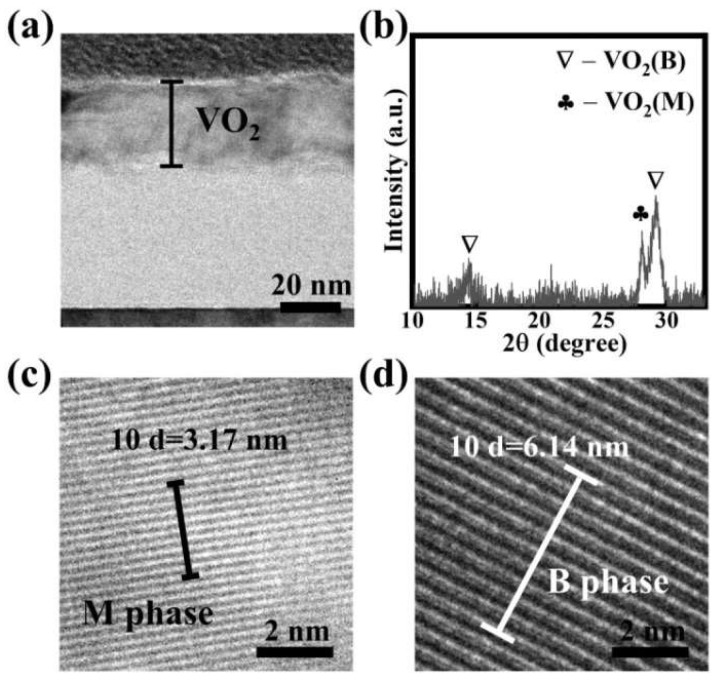
(**a**) TEM cross-section image and (**b**) XRD pattern for the VO_2_ thin films with coexistent VO_2_ (M) and VO_2_ (B) phases, and magnified TEM images for (**c**) VO_2_ (M) and (**d**) VO_2_ (B) phases.

According to previous work [31], we exploited atomic layer deposition and post-annealing processes to obtain coexistent VO_2_ (M) and VO_2_ (B) phases in the VO_2_ thin films. Figure 1a displays a cross-sectional TEM image for a ~32 nm thick VO_2_ thin film. When the XRD measurement was performed on the VO_2_ thin film, diffraction peaks were obvious in the XRD patterns, as shown in Figure 1b. The diffraction peaks near *2θ* = 28.00° were ascribed to monoclinic VO_2_(PDF #09-0142) [29], indicating the presence of a VO_2_ (M) phase in the resulting thin film. In the TEM image (Figure 1c), obvious lattice fringes were also found with a *d-spacing* of *10d* ≈ 3.17 nm, which further confirmed the existence of a VO_2_ (M) phase [32]. As expected for the VO_2_ (M) phase, the VO_2_ (B) phase was also found in the resulting VO_2_ thin film, as shown in Figure 1. XRD peaks near *2θ* = 14.40° and *2θ* = 29.00° in Figure 1b corresponded to VO_2_ (B) (PDF #81-2392) [33], which was also verified by a *d-spacing* of *10d* ≈ 6.14 nm for the lattice fringes in VO_2_ (B) [34] in Figure 1d. All these results suggested the presence of coexistent VO_2_ (M) and VO_2_ (B) phases in the as-prepared VO_2_ thin film.

**Figure 2:**
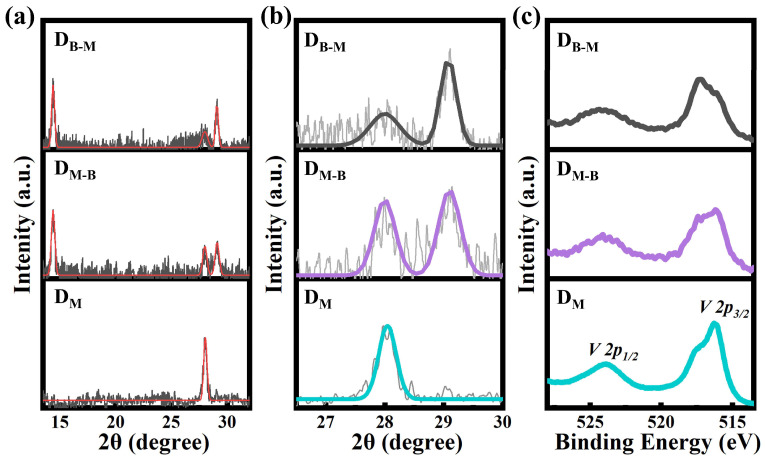
(**a**) XRD patterns, (**b**) fitted XRD patterns, and (**c**) XPS spectra for the D_M_, D_M-B_ and D_B-M_ thin films with coexistent VO_2_ (M) and VO_2_ (B) phases.

As mentioned above, the microstructures of VO_2_ (M) thin films may be changed due to the existence of the VO_2_ (B) phase. Figure 2 displays the microstructure variations in VO_2_ (M) thin films when the content of VO_2_ (B) phases increased in the as-prepared polymorphous VO_2_ thin films. Figure 2a,b displays the XRD patterns of the polymorphous VO_2_ thin films with various VO_2_ (B) contents, referred to as D_M_ (VO_2_ thin film with few VO_2_ (B) phases), D_M-B_ (VO_2_ thin film with some VO_2_ (B) phases) and D_B-M_ (VO_2_ thin film with more VO_2_ (B) phases). The XRD peaks around *2θ* = 28.00° appeared in all D_M_, D_M-B_ and D_B-M_ thin films and were assigned to the (011) plane of VO_2_ (M). Notably, XRD peaks near *2θ* = 14.40° and *2θ* = 29.00° were obvious and found in the D_M-B_ and D_B-M_ thin films, and they were assigned to the (001) and (002) planes in VO_2_ (B) (PDF #81-2392), respectively. For the D_M_ thin film, XRD peaks near *2θ* = 14.40 ° were hardly observed, which indicated the main lattice was the VO_2_ (M) phase in the D_M_ thin film, and few VO_2_ (B) phases might have existed. We also found the intensity ratios at the XRD peaks near *2θ* = 28.00° and *2θ* = 29.00° were different in the case of the D_M-B_ and D_B-M_ thin films. For the D_B-M_ thin film, a larger ratio implied that there was more VO_2_ (B) phases in the VO_2_ thin films with coexistent VO_2_ (M) and VO_2_ (B) phases. Figure 2a suggests that the XRD patterns were different and related to the content of VO_2_ (B) phases. More VO_2_ (B) phases would have enhanced the intensity at about *2θ* = 29.00°, while the intensity declined at about *2θ* = 28.00° for VO_2_ (M). The color changed in the temperature-dependent optical images for the D_M-B_ thin film upon heating (Figure S1 in SI), which indicated that the VO_2_ (M) phase was dominant in the D_M-B_ thin film. In contrast, the invariant color in the optical images (Figure S1 in SI) above room temperature for the D_B-M_ thin film implied that the VO_2_ (B) phase dominated in the D_B-M_ thin film, because the IMT process associated with the VO_2_ (B) phase occurred between 180 K and 300 K. All measurements and analyses above suggested that the contents of the VO_2_ (B) phase in the D_M_, D_M-B_ and D_B-M_ thin films increased gradually.

Subsequently, we fitted the XRD curves and checked the peak variations for the (011)_M_ plane in the VO_2_ (M) phase, as shown in Figure 2b. For the D_M_, D_M-B_ and D_B-M_ thin films, the corresponding *2θ* were about 28.05°, 28.00° and 27.98°, respectively. According to Bragg’s law [35], the *d-spacings* for the (011)_M_ plane in VO_2_ (M) were calculated to be about 3.179 Å, 3.184 Å and 3.186 Å by using the above *2θ* values for the D_M_, D_M-B_ and D_B-M_ thin films, respectively. The *d-spacing* of (011)_M_ plane directly impacted the distance of the V-V lattice along the c_R_ axis, which was related to electron–electron and electron–phonon interactions and the IMT behaviors of VO_2_ (M) [25]. The above results suggested that the VO_2_ (B) phases may affect the lattice, especially the *d-spacing* of the (011)_M_ plane in VO_2_ (M) in the VO_2_ thin films with coexistent VO_2_ (M) and VO_2_ (B) phases. VO_2_ (B) usually formed in an oxygen-excess environment, while VO_2_ (M) formed in an oxygen-deficient environment. An appropriate amount of V could support an environment where VO_2_ (B) was formed [36]. The thin films with more VO_2_ (B) phases annealed at a slower heating rate, resulting in the formation of VO_2_ (B) at a lower temperature and insufficient activation energy to fully convert to stable VO_2_ (R), which would transform to VO_2_ (M) below about 340 K [37]. X-ray photoelectron spectroscopy (XPS) measurements were used to analyze the binding energy and oxidation state related to vanadium [38] (Figure 2c and Figure S2). Figure S2 indicates the fitted peaks for varied oxidation states involving V^+4^ (with binding energies at about 516.2 eV and 523.4 eV) and V^+5^ (with binding energies at about 517.4 eV and 525.2 eV) for the V 2p core-level peaks [38]. We made calculations according to the XPS peaks and found that the valence of V^+4^ was dominant in the D_B-M_ (~57.5%), D_M-B_ (~59.2%) and D_M_ (~67.6%) thin films, while the V^+5^ contents in the D_B-M_ and D_M-B_ thin films were higher than that in the D_M_ thin film. The presence of V^+5^ might have been induced by surface oxidation and adsorption or the precursor of vanadium pentoxide [38]. Noticeably, the binding energy between vanadium atoms had a tendency to decrease from the D_M_ to D_M-B_, and then to D_B-M_ thin films (Figure 2c). As verified in the XRD patterns, the XPS spectra suggested that the increased binding energy possibly corresponded to more VO_2_ (B) existing in the polymorphous thin films. The layered lattice alignments and surface-induced V^+5^ components might have increased the binding energy and stabilized the VO_2_ (B) phases in the D_M-B_ and D_B-M_ thin films [36,39].

**Figure 3:**
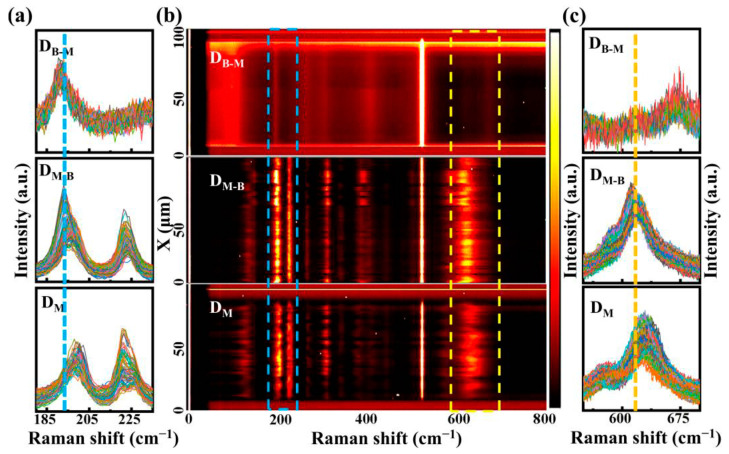
(**a**) Raman spectra at around 194 cm^−1^ (the blue dashed box in (**b**)), (**b**) line-scanned Raman mapping and (**c**) Raman spectra at around 640 cm^−1^ (the yellow dashed box in (**b**)) in the D_B-M_, D_M-B_ and D_M_ thin films. The blue and yellow dashed lines were used to show the position variations of Raman peaks at around 195 and 620 cm^−1^ among the D_B-M_, D_M-B_ and D_M_ thin films, respectively.

Raman spectra were adopted to identify the phases and lattice distortion in the D_M_, D_M-B_ and D_B-M_ thin films, as shown in Figure 3 and Figure S3. The distinguishable Raman shifts at about 137, 195, 224 and 615 cm^−1^ corresponded to the VO_2_ (M) in the D_M_ and D_M-B_ thin films [40], while the one at about 520 cm^−1^ was from silicon substrate. For the D_B-M_ thin film, the Raman vibration modes at around 96, 191, 403 and 668 cm^−1^ were obvious and assigned to VO_2_ (B). The strong Raman peaks (520 cm^−1^) from silicon substrate indicated a deeper detective depth, which implied that the vibration modes of the whole thin film could be obtained along the vertical direction. The vibration mode at about 96 cm^−1^ corresponded to the translations of adjacent layers, and the vibration modes at around 191 and 403 cm^−1^ were related to the bending and stretching of the V-O-V structure in VO_2_ (B) [40]. Generally, the Raman shift at about 195 cm^−1^ (referred as ω_V1_) corresponded to the tilting motion of the V-V dimer, while the one at around 615 cm^−1^ (referred as ω_V-O_) was used to identify the V-O bond vibrations in VO_2_ [41]. Line-scanned Raman mapping in Figure 3 further displays the variations in the phonon vibration modes in the D_M_, D_M-B_ and D_B-M_ thin films. Here, about 100 statistical points were obtained with a scan length of 100 µm and a step of 1 µm during micro-Raman mapping. The thermal effect of the laser could be ignored because no obvious shift was found at 520 cm^−1^ for the silicon substrates [42]. Figure 3a,c show the Raman details at each scan position around 194 cm^−1^ (dashed blue box in Figure 3b) and 640 cm^−1^ (dashed yellow box in Figure 3b) for the D_M_, D_M-B_ and D_B-M_ thin films. In Figure 3a, Raman peaks shift towards low frequencies from around 198.5 cm^−1^ in the D_M_, to around 195 cm^−1^ in the D_M-B_, and then to around 191 cm^−1^ in the D_B-M_ thin film. The Raman peak around 224 cm^−1^ for the VO_2_ (M) phase was observed in both the D_M_ and D_M-B_ thin films, while this peak was hardly observed in the case of the D_B-M_ thin film. As shown in Figure 3c, the ω_V-O_ peak around 668 cm^−1^ was obvious in the D_B-M_ thin film, while the ones around 629 cm^−1^ and 619 cm^−1^ were for the D_M_ and D_M-B_ thin films, respectively. The above peaks indicated the variations in the ω_V1_ and ω_V-O_ modes in the D_M_, D_M-B_ and D_B-M_ thin films. When comparing the varied ω_V1_ and ω_V-O_ modes in the D_M_ to D_M-B_ thin films, we found an obvious shift because of the presence of VO_2_ (B), as shown in Figure 3 and Figure S3. In the D_M_ thin film, the ω_V-O_ peaks were dispersed and changed with the scanning position, while the ω_V-O_ peaks in the D_M-B_ thin film shifted and were concentrated. Meanwhile, the VO_2_ (B) domains were distributed in the D_M-B_ film, which may have induced an abrupt shift [43]. For the D_M_ and D_M-B_ thin films, the variations in the ω_V-O_ modes were different, which may have been attributed to the content or grain size of the VO_2_ (B) phase [43,44] in the corresponding thin films. In addition, the characteristic peaks around 668 cm^−1^ for VO_2_ (B) kept in the D_B-M_ thin film. In general, the phonon behavior in the D_B-M_ thin film was mainly attributed to the VO_2_ (B) phase. All these results further verified the role of VO_2_ (B) in the D_M_, D_M-B_ and D_B-M_ thin films, which were consistent with those indicated during the above XRD measurements.

**Figure 4:**
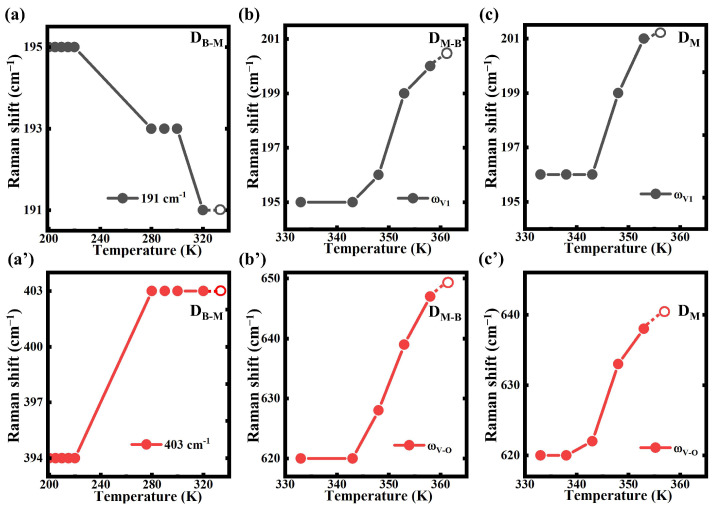
Temperature-dependent Raman modes at around (**a**) 191 and (**a’**) 403 cm^−1^ for the D_B-M_ thin film. Temperature-dependent (**b**) ω_V1_ and (**b’**) ω_V-O_ modes for the D_M-B_ thin film, and temperature-dependent (**c**) ω_V1_ and (**c’**) ω_V-O_ modes for the D_M_ thin film.

As shown in Figure 4 and Figure S4, variable-temperature Raman measurements were taken to detect the thermal-induced structural evolutions in the D_B-M_, D_M-B_ and D_M_ thin films. Figure 4a displays the peak variations in the temperature-dependent Raman spectra for the D_B-M_. The two modes at around ~191 and 400 cm^−1^ were related to the bending and stretching vibration of the V-O-V structure in the VO_2_ (B) phase. When the temperature rose from 220 K to 280 K, the mode at ~394 cm^−1^ shifted to ~403 cm^−1^ (Figure 4a’). The vibration mode around ~195 cm^−1^ at 200 K shifted to ~193 cm^−1^ at 280 K, and then to ~191 cm^−1^ at 320 K. Meanwhile, the intensity of the Raman peak around ~195 cm^−1^ increased obviously from 200 K to 320 K (Figure 4a). There was no significant change in Raman spectra for the D_B-M_ above 320 K. Due to the semimetal nature of the VO_2_ (B) phase at room temperature [44], we could still observe a relatively resolvable Raman shift. In the D_M-B_ thin film, the ω_V1_ vibration mode of ~195 cm^−1^ at 343 K shifted to ~201 cm^−1^ at 358 K, while the ω_V-O_ vibration mode changed from ~620 cm^−1^ to ~647 cm^−1^ due to the thermal-induced lattice distortion. Above 358 K, the ω_V1_ and ω_V-O_ modes vanished due to the formation of metallic rutile VO_2_ during IMT (Figure 4b, b’ and Figure S4b). The metallic rutile phase did not contribute to a discriminable Raman signal, and thus, a flat Raman spectrum indicated its presence. Meanwhile, in the D_M_ thin film, the ω_V1_ vibrations suddenly changed from ~195 cm^−1^ at 343 K to ~201 cm^−1^ at 353 K, and the ω_V-O_ mode at 620 cm^−1^ shifted to 638 cm^−1^. In Figure 4c,c’ and Figure S4c, both two modes disappeared above 353 K, which also resulted from the formation of rutile VO_2_ during IMT. Although the ω_V1_ and ω_V-O_ modes in the D_M-B_ and D_M_ thin films were similar at room temperature, the structural evolution in the D_M-B_ thin film finished at a higher temperature (at 358 K) than that in the D_M_ thin film (at 353 K). We believed that the higher evolution temperature was attributed to the increased content of VO_2_ (B) in the coexistent VO_2_ (M) and VO_2_ (B) thin films. 

**Figure 5:**
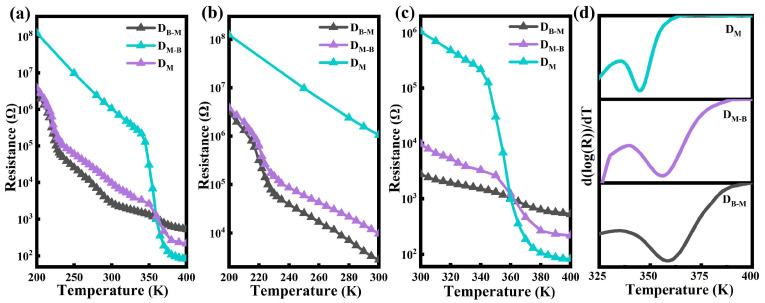
(**a**) Temperature-dependent resistance curves for the D_M_, D_M-B_ and D_B-M_ thin films during a heating process. The resistance–temperature curves in the temperature range of (**b**) 200–300 K and (**c**) 300–400 K, and (**d**) the derivation curves for calculating T_IMT_ for the D_M_, D_M-B_ and D_B-M_ thin films.

As indicated by the XRD and Raman measurements, the coexistent VO_2_ (M) and VO_2_ (B) phases were likely to modify the microstructure and the temperature-dependent structure evolutions. Subsequently, the temperature dependent resistance of the D_M_, D_M-B_ and D_B-M_ thin films is shown in Figure 5 and Figure S5. The resistance of the D_B-M_ and D_M-B_ thin films changed and underwent a change of over 2 orders of magnitude when the temperature went from 200 K to 300 K, which was related to the IMT process of the VO_2_ (B) phase [27]. However, no such decrease occurred in the D_M_ thin film, as displayed in Figure 5b. The following drop in the resistance of the D_M_, D_B-M_ and D_M-B_ thin films at around 350 K was related to VO_2_ (M) (Figure 5c) [45]. For the D_M-B_ thin film, the small change in resistance from 200 K to 300 K shown in Figure 5b may have resulted from the lower VO_2_ (B) content and the poorer crystallinity [32]. Figure 5c further suggests the variations in resistance changed from 300 K to 400 K in the D_M_, D_M-B_ and D_B-M_ thin films [46]. Notably, the increased content of VO_2_ (B) would have greatly reduced the resistance variations in the as-prepared coexistent VO_2_ (M) and VO_2_ (B) thin films from 300 K to 400 K [29]. By taking the derivation curves and using a Gaussian fit, we obtained the transition temperature (T_IMT_) above room temperature of about 345, 357 and 360 K for the D_M_, D_M-B_ and D_B-M_ thin films, respectively (Figure 5d). As we obtained in the temperature-dependent Raman spectra, the increased contents of VO_2_ (B) led to an increase in T_IMT_ [44]. In addition, the T_IMT_ increased with the widened plane spacing from the D_M_ to D_M-B_ and then to D_B-M_ thin films. These results implied that coexistent polymorphs and lattice distortion accompanied and caused such changes in the VO_2_ thin films with the coexistent VO_2_ (M) and VO_2_ (B) phases [45].

## 3. Conclusions

We have demonstrated the lattice variations and phase-dependent IMT behaviors in the polymorphous thin films with coexistent VO_2_ (M) and VO_2_ (B). XRD and Raman measurements revealed that the increased (011)_M_ plane spacing and the red-shifted V-V and V-O vibrations in VO_2_ (M) were triggered by the presence of more VO_2_ (B) phases. Temperature-dependent electrical and Raman analyses indicated that increased VO_2_ (B) phases would promote the transition temperature and reduce the strength of IMT in the polymorphous VO_2_ thin films. We believe that such a study will contribute to uncovering the coexistent polymorphous phases and the underlying physics behind IMT in VO_2_ towards advanced electronic and optical devices.

## 4. Experimental Section

The polymorphous VO_2_ thin films were obtained with a combination of ALD and post-annealing procedures according to the processes previously reported [31]. The temperature-dependent optical images were obtained via a optical microscope with a home-made heating stage. Raman spectra (Nanofinder 30, Tokyo Instruments, Inc., Tokyo, Japan, the spot size of laser was about 500 nm, power was 1 mW and the grating was 1800 g/mm), micro-XRD (Bruker D8 Discover, Bruker, Karlsruhe, Germany), XRD (Bruker D8, Bruker, Karlsruhe, Germany), X-ray photoelectron spectroscopy (XPS, ESCALAB Xi+, Thermo Fischer Scientific Inc., Waltham, MA, USA) and transmission electron microscopy (TEM, JOEL JEM-2100F, JOEL Ltd., Tokyo, Japan) were used to characterize the VO_2_ thin films. Electrical curves were recorded at variable temperatures from 77 K to 450 K on a cryogenic probe station (Lakeshore TTPX, Lake Shore Cryotronics, Inc., Woburn, MA, USA) with a semiconductor characterization system (Keithley 2636B, Tektronix, Inc., Beaverton, OR, USA).

## Data Availability

Not applicable.

## Supplementary Materials

The following supporting information can be downloaded at: https://www.mdpi.com/article/10.3390/nano13091514/s1, Figure S1: Optical microscopy images for the D_M-B_ (a) and D_B-M_ (b) thin films at varied temperatures, Figure S2: (a) The V 2p and O 1s XPS spectra and (b) the fitted V 2p XPS spectra for the D_M_, D_M-B_ and D_B-M_ thin films with coexistent VO_2_ (M) and VO_2_ (B) phases, Figure S3: (a) Raman spectra for the polymorphous VO_2_ thin films with coexistent VO_2_ (M) and VO_2_ (B) phases, and details at around 640 cm^−1^ for the D_B-M_ (b), D_M-B_ (c) and D_M_ (d) thin films, Figure S4: Temperature-dependent Raman spectra for the D_B-M_ (a), D_M-B_ (b) and D_M_ (c) thin films, Figure S5: Temperature-dependent resistance curves and their derivative curves for the D_B-M_ (a), D_M-B_ (b) and D_M_ (c) thin films.
