# Supplementary material for: Coexistent VO2 (M) and VO2 (B) Polymorphous Thin Films with Multiphase-Driven Insulator–Metal Transition
Source: Nanomaterials (Basel). 2023 Apr 28;13(9):1514. doi: 10.3390/nano13091514 (PMC10180398; doi:10.3390/nano13091514)
Supplement: Supplementary file 1 [file nanomaterials-13-01514-s001.zip › nanomaterials-2367604-supplementary.pdf]

nanomaterials

Supplementary Materials

Coexistent VO_2 (M) and VO_2 (B) polymorphous thin films with multiphase-driven insulator-metal transition

Mengxia Qiu, [ab] **Wanli Yang,** [c] **Peiran Xu,** [a] **Tiantian Huang,** [a] **Xin Chen,**[*ac] **Ning Dai** [*abc]

[a]State Key Laboratory of Infrared Physics, Shanghai Institute of Technical Physics, Chinese Academy of Sciences, Shanghai 200083, China. Email: xinchen@mail.sitp.ac.cn, ndai@mail.sitp.ac.cn
[b]School of Physical Science and Technology, ShanghaiTech University, Shanghai 201210, China
[c]Hangzhou Institute for Advanced Study, University of Chinese Academy of Sciences, Hangzhou 310024, China

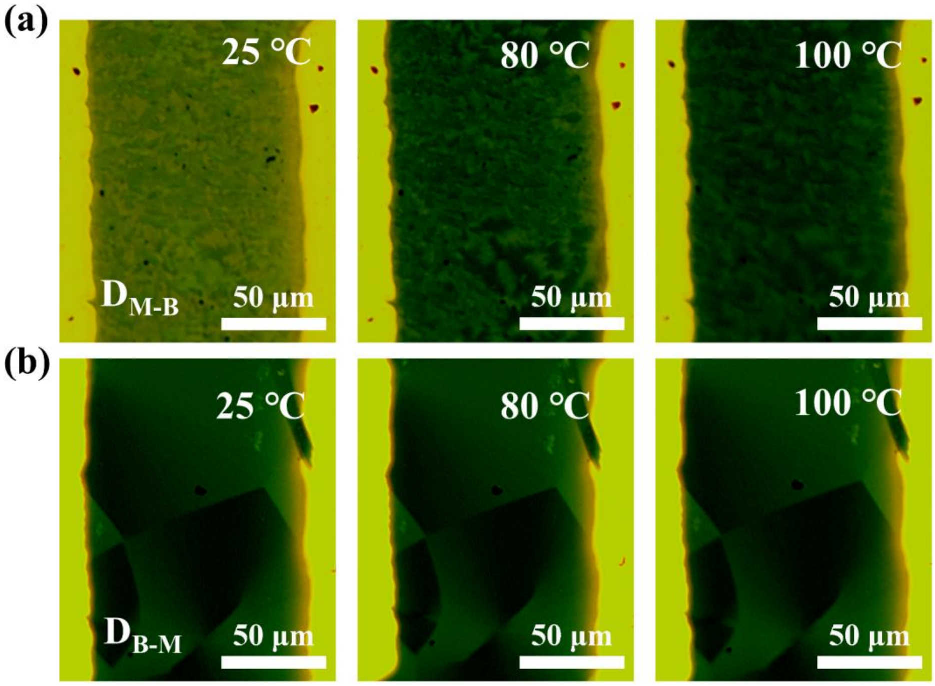

Figure S1 Temperature-dependent optical microscopy images for the D$_{M-B}$ (a) and D$_{B-M}$ (b) thin films at varied temperature.

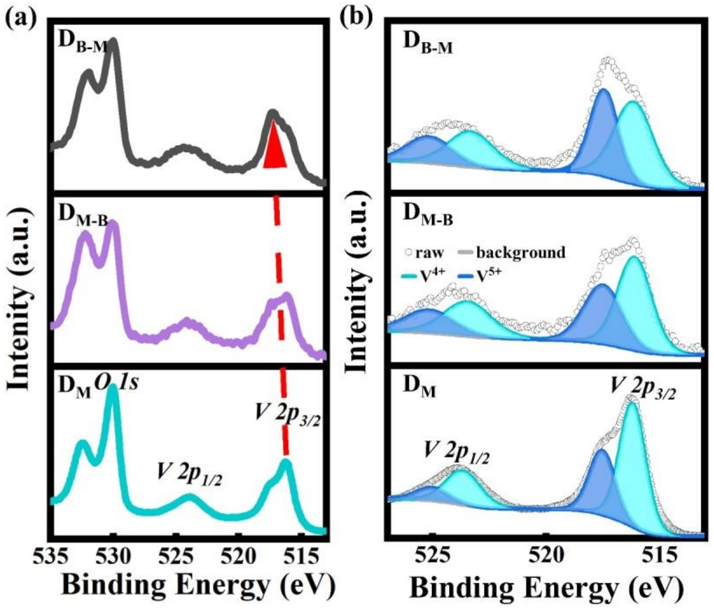

Figure S2 (a) The V 2p and O 1s XPS spectra and (b) the fitted V 2p XPS spectra for the D_M, D_{M-B} and D_{B-M} thin films with coexistent VO_2 (M) and VO_2 (B) phases.

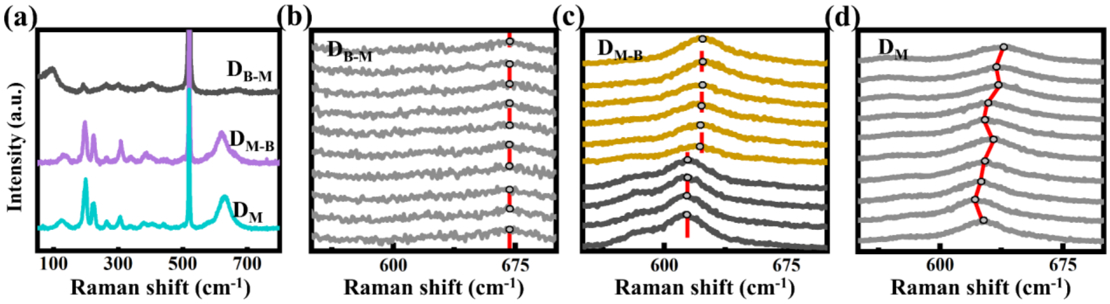

Figure S3 a) Raman spectra for the polymorphous VO_2 thin films with Coexistent VO_2 (M) and VO_2 (B) phases, and details at around 640 cm^{-1} for the D_{B-M} (b), D_{M-B} (c) and D_M (d) thin films.

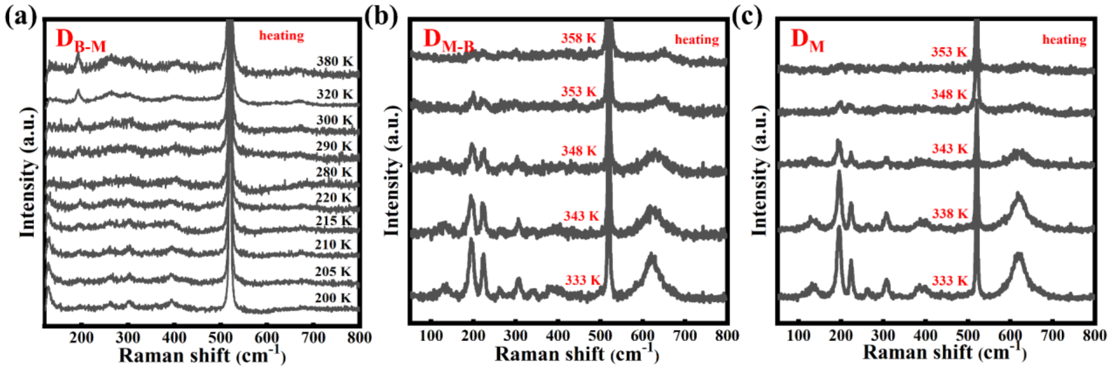

Figure S4 Temperature-dependent Raman spectra for the $D_{B\text{-}M}$ (a), $D_{M\text{-}B}$ (b) and D_M (c) thin films.

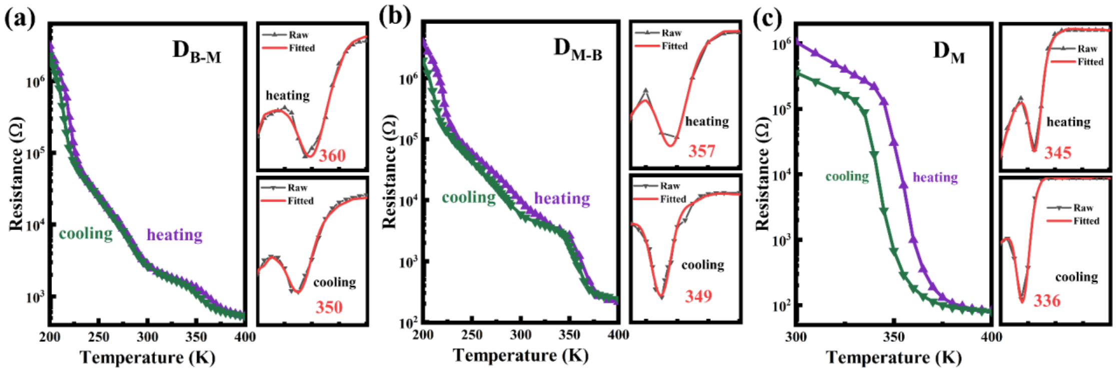

Figure S5 Temperature-dependent resistance curves and their derivative curves for the D_{B-M} (a), D_{M-B} (b) and D_M (c) thin films.
